# Prevalence, factors associated and management of needle phobia among the general population in Saudi Arabia and Egypt

**DOI:** 10.1186/s12888-024-05757-5

**Published:** 2024-05-14

**Authors:** Hassan Alwafi, Abdallah Y. Naser, Nada A. Alsaleh, Jamila Kamal Asiri, Rawan M. Almontashri, Lujain Mohammed Alqarni, Rawan Sulaiman Salawati, Alaa Alsharif, Abdulelah M. Aldhahir, Abdullah A. Alqarni, Waleed Hafiz, Jaber S. Alqahtani, Emad Salawati, Mohammed A. Almatrafi, Mohamed Bahlol

**Affiliations:** 1 Department of Pharmacology and Toxicology, Faculty of Medicine, Umm Al-Qura University, 8WMR+23J Mecca, Al Abdeyah, Alawali, Mecca Saudi Arabia; 2 Department of Applied Pharmaceutical Sciences and Clinical Pharmacy, Faculty of Pharmacy, Isra University, Amman, Jordan; 3 Department of Pharmacy Practice, Faculty of Pharmacy, Princess Nourah bint Abdulrahman University, Riyadh, Kingdom of Saudi Arabia; 4 Faculty of Medicine, Umm Al-Qura University, Makkah, Saudi Arabia; 5 Respiratory Therapy Department, Faculty of Applied Medical Sciences, Jazan University, Jazan, Saudi Arabia; 6 Department of Respiratory Therapy, Faculty of Medical Rehabilitation Sciences, King Abdulaziz University, Jeddah, Saudi Arabia; 7 Department of Internal Medicine, Faculty of Medicine, Umm Al-Qura University, Makkah, Saudi Arabia; 8 Department of Respiratory Care, Prince Sultan Military College of Health Sciences, Dammam, Saudi Arabia; 9 Department of Family Medicine, Faculty of Medicine, King Abdulaziz University, Jeddah, Saudi Arabia; 10 Department of Pediatrics, Faculty of Medicine, Umm Al-Qura University, Makkah, Saudi Arabia; 11 Speciality of Pharmaceutical Management and Economics, Department of Pharmacy Practice and Clinical Pharmacy, Faculty of Pharmacy, Egyptian Russian University, Cairo, Egypt

**Keywords:** Anxiety, Needle phobia, Fear, Saudi Arabia, Egypt, Survey

## Abstract

**Objective:**

This study aims to assess the prevalence of needle phobia among Saudi and Egyptian adult populations. In addition, underlying causes and strategies that can be utilized to address needle fear were investigated.

**Methods:**

A cross-sectional online survey study was conducted in Saudi Arabia and Egypt between 1 May and 30 June 2023. Participants aged 18 years and above and living in Saudi Arabia and Egypt were eligible to complete the survey. Participants were invited to participate in this study through social media platforms (Facebook, X, Snapchat, and Instagram). A convenience sampling technique was used to recruit the study participants. A 21-item questionnaire consisting of four sections including a Likert scale score was used to answer the research objectives. Numeric data were presented as mean ± SD. For categorical variables, percentages were used. Comparison between groups were made by Student’s t-test or Mann Whitney test according to data distribution. Chi squared tests for categorical values were conducted. A binary logistic regression analysis was conducted to investigate factors associated with needle phobia.

**Results:**

A total of 4065 participants were involved in this study (Saudi Arabia: 2628 and Egypt: 1437). Around one-third of the study participants (36.5%) confirmed that they have needle phobia. Most of the study participants (81.1%) reported that they have had needle phobia since they were under 18 years of age. Pain, general anxiety, and fear of making a mistake during the procedure were the most commonly reported contributors for fear of needles during or before a medical procedure. Around 15.8% of the study participants reported that they have tried to get rid of phobia from needles. Non-surgical alternatives (such as oral medications and patches) and using smaller/thinner needles were the most commonly reported interventions that reduced fear of needles. Binary logistic regression analysis identified that females, those who are aged (41–50 years), widowed, those with bachelor’s degrees and higher education, and those unemployed were more likely to have needle phobia compared to others.

**Conclusion:**

Our study highlighted the high prevalence of needle fear within an adult population in Egypt and Saudi Arabia. Females, those who are aged (41–50 years), those widowed, those with higher education degrees, those unemployed, those working in the health sector and people with low income were more likely to have needle phobia compared to others.

## Background

Injections are commonly used in daily medical practices and for multiple indications including drugs, vaccines and other substances to be delivered into the body, or to extract fluids and tissues for medical diagnosis [1]. Anxiety is a major concern for public health, and it can be a major limitation during medical procedures and interventions, including needles interventions [2]. Anxiety that is out of proportion to the threat in the environment and the event is referred to as needle phobia according to the Fifth Edition of the Diagnostic and Statistical Manual of Mental Disorders (DSM-5) [3]. Our genetics, environments, and past experiences could all contribute to needle phobia. However, needle phobia is associated with anxiety and fear, and avoidance behaviour is common among those patients, which can negatively impact the medical treatment and patient outcomes [2, 4]. There is a spectrum of needle phobia severity, and it can result in delayed therapy, treatment avoidance, immunization reluctance, and psychological, social, and physiological repercussions [5]. Treating needle phobia has been given a relatively low priority, unfortunately, since it is rare that the patient’s avoidance will be life threatening. However, in recent times, when the whole world was facing the COVID-19 pandemic, vaccinations became the forefront of containing and reducing the severity of the disease. Patients with needle phobia would often refuse vaccinations, and this would subsequently put the general population at risk. This proves that treating needle phobia should be given a higher priority than what it receives currently. Various studies were conducted with different strategies and different approaches to assess needle phobia [6–9]. Nonetheless, there may be some possible limitations, as they only evaluated a single strategy without comparing other strategies or approaches to assess needle phobia and had issues with research samples and selection [6]. In addition, previous studies that investigated needle phobia in the Middle East and Arab countries are limited.

Saudi Arabia and Egypt are two of the highly populated countries in the Arab world and they share similar religious and cultural influencing characteristics. Therefore, this study aims to assess the prevalence of needle phobia among Saudi and Egyptian adult populations. In addition, underlying causes of needle phobia and strategies that can be utilized to address needle fear was investigated.

## Methods

### Study design

A cross-sectional online survey study was conducted in Saudi Arabia and Egypt between 1st of May and 30 June 2023.

### Study population

Participants aged 18 years and above and living in Saudi Arabia and Egypt were eligible to complete the survey. Participants were invited to participate in this study through social media platforms (Facebook, X, Snapchat, and Instagram). The study samples were invited using a survey link, which was posted and then distributed every week for a consecutive of eight weeks to help in targeting the study population. All participants voluntarily participated in the study. The study aims and objectives were clearly explained at the beginning of the invitation letter of the survey.

### Sampling strategy

A convenience sampling technique was used to recruit the study participants.

### Questionnaire tool

This study survey was used in a previously validated questionnaire. The original questionnaire was validated and developed based on a comprehensive literature review that investigated gaps in the knowledge, underlying causes of needle phobia, and variations in the prevalence of need phobia. The objectives and orientation of the questionnaire were to the global adult population [6]. The questionnaire was translated into Arabic using the forward–backward method and then used in this study. Using a pre-existing survey has the advantage of being validated, and therefore, increasing the validity and reliability of the study. However, it also allows for comparison with different populations [10]. A 21-item questionnaire was used in this study (attached in the supplements). The questionnaire consisted of four main sections and included multiple-choice, 11-point Likert-like scale, ranking, and open-ended questions. The first section included background questions regarding demographics and overall perception of medical care. The second and third sections assessed how common needle phobia is, its underlying reasons, and its impacts on overall well-being. The fourth section covered mitigation strategies to identify potential approaches that may be used to alleviate the fear of needles.

### Sample size determination

To estimate the sample size, we used the WHO recommendations for the minimal sample size needed for a prevalence study [11]. Using a confidence interval of 95%, a standard deviation of 0.5, and a margin of error of 5%, the required sample size was 385 participants from each study population.

### Ethical statement

The Research Ethics Committee approved this study at Umm Al Qura University, College of Medicine. The study participants were briefed about the objectives of the study. All participants gave an informed consent.

### Statistical analysis

SPSS software, version 26 was used for statistical analyses. Numeric data were presented as mean ± SD. For categorical variables, percentages were used. Comparison between groups were made by Student’s t-test or Mann Whitney test according to data distribution. Chi squared test for categorical values were conducted. A binary logistic regression analysis was conducted to investigate factors associated with needle phobia. The independent variables in the regression model were participants’ demographic characteristics, and the dependent variable was the reporting of needle phobia. The odds ratio with 95% confidence interval was presented to demonstrated the strength of the association between dependent and independent variables. The significance level was assigned as p-value less than 0.05.

## Results

### Participants’ demographic characteristics

A total of 4065 participants were involved in this study (Saudi Arabia: 2628 and Egypt: 1437). The majority of them were females (76.2%), aged 18–23 years (64.8%), and single (80.2%). More than half of them (61.8%) reported that they hold bachelor’s degree and were university students (55.3%). Almost half of the study sample (50.9%) reported that their monthly income category is less than 1500$. Table 1 presents participants’ demographic characteristics.

**Table 1:** Participants’ demographic characteristics

Variable	Overall (*n* = 4065)		Saudi Arabia (*n* = 2628)		Egypt (*n* = 1437)	
	Frequency	Percentage	Frequency	Percentage	Frequency	Percentage
**Gender**						
Females	3098	76.2%	2220	84.5%	878	61.1%
**Age categories**						
18–23 years	2636	64.8%	1635	62.2%	1001	69.7%
24–30 years	861	21.2%	677	25.8%	184	12.8%
31–40 years	244	6.0%	166	6.3%	78	5.4%
41–50 years	199	4.9%	87	3.3%	112	7.8%
51–60 years	104	2.6%	51	1.9%	53	3.7%
61 years and over	21	0.5%	12	0.5%	9	0.6%
**Marital status**						
Single	3261	80.2%	2149	81.8%	1112	77.4%
Married	690	17.0%	394	15.0%	296	20.6%
Divorced	83	2.0%	71	2.7%	12	0.8%
Widowed	31	0.8%	14	0.5%	17	1.2%
**Education level**						
High school or lower	792	19.5%	612	23.3%	180	12.5%
Bachelor’s degree	2511	61.8%	1845	70.2%	666	46.3%
Higher education	762	18.7%	171	6.5%	591	41.1%
**Employment status**						
Retired	107	2.6%	73	2.8%	34	2.4%
Unemployed	798	19.6%	512	19.5%	286	19.9%
Work in healthcare sector	444	10.9%	306	11.6%	138	9.6%
University student	2249	55.3%	1482	56.4%	767	53.4%
Work outside healthcare sector	467	11.5%	255	9.7%	212	14.8%
**Monthly income categories**						
Less than 1500$	2068	50.9%	872	33.2%	1196	83.2%
1500–3000$	667	16.4%	512	19.5%	155	10.8%
3000–4500$	448	11.0%	411	15.6%	37	2.6%
4500–6000$	402	9.9%	372	14.2%	30	2.1%
6000$ and over	480	11.8%	461	17.5%	19	1.3%

### Needle phobia profile

Table 2 presents the study participants’ needle phobia profile. Around one-third of the study participants (36.5%) confirmed that they have non-needling (needle-free) medical concerns. A similar proportion of the participants (35.4%) confirmed that they have a needle phobia, of which 18.8% reported that they experienced it during the medical procedure. Around 15.3% of the participants reported that they have the condition(s) that frequently require injections and blood draws. The study participants rated their pain tolerance as being moderate (mean tolerance scale is 6.4 (SD: 2.4) out of 10). Most of the study participants (81.1%) reported that they have needle phobia since they were under 18 years old. Around 14.2% of the study participants reported that they have a family member(s) who is/are diagnosed with needle phobia. The self-rated severity of needle phobia during, before or after medical procedures among the study participants was low (the mean score is 3.7 (SD: 2.9)). Pain, general anxiety, and fear of making a mistake during the procedure were the most commonly reported contributors for fear of needles during or before a medical procedure. Around one-fifth of the study participants (22.2%) reported that they avoided a medical treatment (e.g., blood draws, injections, or vaccinations) before because they knew there was a needle involved. Similarly, the main reasons that made them avoid this procedure were Pain, general anxiety, and fear of making a mistake during the procedure. The vast majority of the participants (82.6%) reported that they would have the treatment (e.g. blood draws, injections or vaccinations) if there were no needles.

**Table 2:** Needle phobia profile

Variable	Frequency (percentage)
**Do you have any non-needling medical concerns?**	
Yes	1484 (36.5%)
**Do you have needle phobia?**	
Yes	1440 (35.4%)
**If yes, when did you experience this?** (You can choose more than one answer)	
Before a medical procedure	623 (15.3%)
During a medical procedure	763 (18.8%)
After a medical procedure	223 (5.5%)
**Do you have any conditions that frequently require injections and blood draws?**	
Yes	621 (15.3%)
**On a scale from 0 to 10, how would you rate your pain tolerance in general?** (mean (SD))	6.4 (2.4)
**If yes, how long have you been suffering from needle phobia?** (*n* = 1358)	
Under 18 years old	1101 (81.1%)
Over 18 years old	257 (18.9%)
**Has any family member been diagnosed with or reported to have needle phobia?**	
Yes	576 (14.2%)
**Please rate the severity of your fear of needles during, before or after medical procedures from zero to ten where zero is (no fear at all) and ten is (extreme fear or avoidance of the procedure)** (mean (SD))	3.7 (2.9)
**Which of the following contributes to your fear of needles during or before a medical procedure?** (Please select all that apply)	
Pain	1520 (37.4%)
General anxiety	1428 (35.1%)
Fear of making a mistake during the procedure	1288 (31.7%)
Previous traumatic experience	654 (16.1%)
Fear of seeing blood	396 (9.7%)
Disgust about the procedure	334 (8.2%)
Fear of fainting/feeling dizzy	329 (8.1%)
**Have you ever avoided a medical treatment** (e.g.: blood draws, injections, or vaccinations) **because you knew there was a needle involved?**	
Yes	903 (22.2%)
**If yes, what is the main reason that made you avoid this procedure?** (*n* = 873)	
General anxiety	274 (31.4%)
Pain	188 (21.5%)
Fear of making a mistake during the procedure	128 (14.7%)
Previous traumatic experience	117 (13.4%)
Fear of seeing blood	74 (8.5%)
Disgust about the procedure	47 (5.4%)
Fear of fainting/feeling dizzy	45 (5.2%)
**If yes, would you have treatment** (e.g. blood draws, injections or vaccinations) **if there were no needles?** (*n* = 873)	
Yes	721 (82.6%)
**Which of the following actions should you avoid to reduce your exposure to needles?** (You can choose more than one answer)	
Blood donation	1067 (26.2%)
Draw blood from a vein in the arm	1050 (25.8%)
An injection for pain relief	972 (23.9%)
Vaccinations	746 (18.4%)
An injection for a mild medical condition (low risk of disease)	614 (15.1%)
Taking blood from capillaries (finger prick)	504 (12.4%)
An injection to treat a severe medical condition (at risk of illness or death)	371 (9.1%)

### Strategies to reduce needle phobia

Table 3 below presents study participants’ strategies to reduce needle phobia. Around 15.8% of the study participants reported that they have tried to get rid of their phobia of needles. Nurses were the most commonly reported medical staff members with whom the participants shared their fear of needles. The response of the service provider was described to be moderate (mean score of 6.0 (SD: 2.9). Non-surgical alternatives (such as oral medications and patches) and using smaller/thinner needles were the most commonly reported interventions that reduce fear of needles. In addition, relaxation techniques (i.e., deep breathing) and using topical anaesthetic creams were reported to reduce participants’ fear of needles.

**Table 3:** Strategies to reduce needle phobia

Variable	Frequency (percentage)
**Have you ever sought help to get rid of your phobia of needles?** (*n* = 3708)	
Yes - I have seen a personal therapist	290 (7.8%)
Yes - I saw a remote handler (ex: video call, phone call or messages)	198 (5.3%)
Yes - other procedures	97 (2.6%)
No	3123 (84.2%)
**Have you shared your fear of needles with any of the medical staff below?** (*n* = 3708)	
Nurse	486 (13.1%)
Doctor	300 (8.1%)
Another health care professional	65 (1.8%)
**If yes for the first question, please describe the response of the service provider on a scale of 0 to 10, where 0 is “not helpful” and 10 is “very helpful”** (mean (SD))	6.0 (2.9)
**Would any of the following interventions based on your fear of needles reduce your fear of needles? Please specify all that apply and are effective in addressing your fear of needles**:	
Non-surgical alternatives (such as oral medications and patches)	1760 (43.3%)
Smaller/thinner needles	1408 (34.6%)
Automated syringes (i.e., invisible needles)	706 (17.4%)
Needle-free syringes (drugs and vaccines delivered intramuscularly or under the skin through a narrow, fine stream instead of a needle)	596 (14.7%)
Insulin delivery devices (such as single-use pens and insulin pumps)	366 (9.0%)
**Would any of the following interventions reduce your fear of needles?** Please specify all that apply and are effective in addressing your fear of needles	
Relaxation techniques (i.e., deep breathing)	987 (27.9%)
Use topical anaesthetic creams	816 (23.1%)
Education/explaining how medical equipment works	582 (16.5)
Distractions during the procedure (e.g., watching TV, virtual reality (VR) headset)	519 (14.7%)
Consult with a physician regarding the importance of the procedure/treatment	315 (8.9%)
See a therapist (e.g., cognitive behavioural therapy)	190 (5.4%)
Watch blood draw videos before/during the procedure	127 (3.6%)

### Predictors of needle phobia

Binary logistic regression analysis identified that females, those who are aged (41–50 years), widowed, those with bachelor’s degree and higher education, and unemployed were more likely to have needle phobia compared to others (*p* < 0.05), Table 4.

**Table 4:** Predictors of needle phobia

Variable	Odds ratio of having needle phobia	95% confidence interval
**Gender**		
Females (Reference category)	1.00	
Males	0.53 (0.45–0.62)	*p* < 0.001***
**Age categories**		
18–23 years (Reference category)	1.00	
24–30 years	0.90 (0.76–1.05)	0.172
31–40 years	0.97 (0.74–1.28)	0.843
41–50 years	1.35 (1.01–1.81)	0.041*
51–60 years	0.88 (0.58–1.34)	0.555
61 years and over	0.57 (0.21–1.55)	0.271
**Marital status**		
Single (Reference category)	1.00	
Married	1.13 (0.096–1.34)	0.148
Divorced	1.27 (0.82–1.98)	0.287
Widowed	2.23 (1.10–4.53)	0.027*
**Education level**		
High school or lower (Reference category)	1.00	
Bachelor’s degree	0.88 (0.77-1.00)	0.047*
Higher education	1.35 (1.15–1.58)	*p* < 0.001***
**Employment status**		
Retired (Reference category)	1.00	
Unemployed	1.28 (1.10–1.50)	0.002**
Work in healthcare sector	0.75 (0.60–0.93)	0.008**
University student	0.98 (0.86–1.12)	0.757
Work outside healthcare sector	0.91 (0.75–1.12)	0.386
**Monthly income categories**		
Less than 1500$ (Reference category)	1.00	
1500–3000$	0.94 (0.79–1.12)	0.519
3000–4500$	0.84 (0.68–1.04)	0.100
4500–6000$	0.90 (0.73–1.12)	0.356
6000$ and over	0.72 (0.58–0.88)	0.002**

## Discussion

This cross-sectional study estimated the prevalence of needle phobia in Saudi Arabia and Egypt. Additionally, we sought to identify the underlying causes contributing to needle fear and explore potential strategies to effectively address this prevalent issue. Around one-third of the participants, 35% confirmed having needle phobia, with almost 19% experiencing it during medical procedures. In 2019, in a systematic review that analyzed 119 research papers from different countries, the authors concluded that needle phobia were around 20–30% in adults [1]. However, it is important to highlight that there is a wide variation in the literature regarding the prevalence of needle phobia, with most of the studies reporting a prevalence rate between 3% and 30% [12, 13], which may be due to heterogeneity in the definition of needle phobia and needle fear [13]. However, in the Middle East, similar results were also reported in a recent study in Jordan that involved around 1182 Jordanians [14]. Our study found that the majority of people who experienced needle fear and needle phobia were female (77%). Similar results were also found in the literature, indicating that needle fear was particularly high in Saudi Arabia [1].

Needle phobia can be related to several factors including genetics, environmental, and previous experiences [13]. In this study, approximately 16% of people who experienced needle phobia reported a frequent need for injections and blood draws, possibly contributing to the exacerbation of their needle phobia. Similarly, 14% of individuals with needle phobia have family members who share similar concerns, hinting at a potential hereditary or learned component to this fear. However, there’s clear evidence that needle phobia has a hereditary component. Also, based on earlier studies which support the hypothesis that needle fear is a trait that can be a combination of both inherited and learned [15]. Associative fear of needles or witnessing a traumatic experience of members of the family may also lead to psychological symptoms including unexplained anxiety and panic attacks [16]. However, other factors, such as cultural factors, may also contribute to the prevalence of needle phobia, as it may influence the attitude and perception of individuals towards medical procedures and interventions [17]. In some cases, religious factors may also influence the acceptance and fear of needles and other medical interventions, such as blood transfusion and surgery [17].

A substantial majority, constituting 81.1%, reported experiencing needle phobia since their time under the age of 18. During childhood, fears are often transient phenomena, but they occasionally turn into phobias. Also, needle phobia could be diagnosed in 19% of children aged 4–6 years, and it could be reduced with age [18]. The average pain tolerance within this population is moderate, as indicated by a mean tolerance scale of 6.4 (SD: 2.4) out of 10, suggesting that the fear of needles may not solely be attributed to an exceptionally low pain threshold. The most prevalent trigger for needle-related fear is associated with medical procedures. Alarmingly, 22.2% of participants in the study reported actively avoiding medical treatments, such as blood draws, injections, or vaccinations, due to their apprehension surrounding needles. This avoidance behavior underscores the significant impact of needle phobia on healthcare decisions and adherence to necessary medical procedures [18].

In terms of the severity of needle phobia, participants self-rated it as relatively low, with a mean score of 3.7 out of 10. Approximately one-fifth of the participants reported avoiding medical treatments involving needles due to various factors such as pain, general anxiety, and fear of making a mistake during the procedure. However, the majority of participants stated that they would be willing to undergo treatments if needles were not involved. Specifically, blood donation and drawing blood from a vein in the arm were the procedures most commonly avoided. Results of the prevalence of avoidance due to fear was 27% in hospital employees, 18% in healthcare workers at long-term care facilities, which was similar to previous reports [1, 7].

Binary logistic regression analysis revealed that certain factors were associated with a higher likelihood of needle phobia. These factors included being female, aged between 41 and 50 years, being widowed, having higher education, and being unemployed. Similarly, previous studies conducted in Jordan and India showed that needle phobia was more common among females than males [14, 19, 20]. Potential causes for the higher susceptibility of females to needle phobia include variations in pain perception or anxiety levels [21]. Individuals aged 41 to 50 may possess a greater quantity of exposure to needle-based medical procedures, which could elevate their propensity to develop needle phobia. Widowhood may be correlated with increased levels of tension or diminished social support, both of which may contribute to needle-related anxiety. An increased consciousness regarding the risks associated with needles may result from pursuing higher education, thereby exacerbating needle phobia [22]. Unemployment may be associated with elevated levels of tension or anxiety, which may intensify apprehensions concerning needles. It is plausible that these variables, in conjunction with personal dispositions and prior encounters, exert an impact on the formation of needle phobia.

The reported prevalence of needle phobia in the Middle east countries ranged between 2.9% and 27.4% [6, 14]. Education regarding procedures, relaxation techniques such as deep breathing, and distractions like music are all components of needle phobia management. Cognitive behavioural therapy, topical anaesthetics, and gradual needle exposure may also be beneficial. Hypnosis and participation in support groups or therapy are further approaches that can be employed to mitigate the anxiety that may accompany medical procedures [23, 24].

Our study shows that 84% of participants never sought help to address their fear of needles. However, the majority of the participants shared this fear with nurses and other healthcare providers. The nurse plays a vital role in alleviating needle-related distress and phobia and distress [24]. However, different approaches aimed at alleviating needle fear were identified in the literature and examined in our study to explore patient preferences and perspectives on their effectiveness. In our study, non-surgical alternatives such as oral medications, patches, followed by smaller or thinner needles, relaxation techniques, and topical anesthetic creams were reported as the most common interventions that helped reduce the fear of needles. Consistent with our findings, an international study conducted by Alsbrooks and Hoerauf (2022) reported that non-invasive alternatives, smaller needles, relaxation techniques and applying local anesthetic creams were identified as the most helpful interventions in alleviating needle fear [6]. Two randomized controlled trials (RCTs) demonstrated the effectiveness of smaller or thinner needles in effectively reducing injections-related pain, which is closely associated with needle phobia [25, 26]. Moreover, RCTs have demonstrated the positive effects of non-invasive alternatives on both pain reduction and patient satisfaction [27, 28].

The providers were rated with a perceived helpfulness of 6 on a scale ranging from 0 to 10. However, this moderate rating was based on a relatively small percentage of the participants. However, this also shows that needle phobia is not addressed as it should be, healthcare workers should provide information and motivation to the affected, so that they seek medical advice and manage this problem. Educating healthcare workers regarding this issue would help them identify needle phobia and subsequently, manage it. A similar pattern can be seen in Alsbrook et al.’s (2022) study [6].

Our study demonstrated that there is a significant relationship between low income and needle phobia; this is supported by Milgrom et al.’s (1995) study which shows that the main reason behind refusal of dental procedures in low income American families were the use of needles [15].

Medical practitioners should clearly and patiently explain the procedure to patients and address their queries and worries, especially when dealing with the child age group. Distracting children by putting child-friendly posters and televisions showing cartoons in the waiting room and clinic can reduce the anxiety that the child faces [29, 30]. Keeping the needles outside the field of vision of the patient can also be helpful in reducing stress levels. Implementing these strategies will have a remarkable effect on the patient, as troublesome and traumatic encounters with needles often result in patients developing needle phobia [2, 6]. In our study, 14.7% agreed that these strategies can be useful while tackling needle phobia.

Our study highlighted the use of relaxation techniques, behavioral therapy, topical anesthetics and other non-psychotropic interventions to treat needle phobia. This is supported by Love et al.’s (2021) study, showing that psychotropic interventions like administering benzodiazepines have been ineffective in controlling needle phobia and have made symptoms worse over time [13].

### Strength and weakness

This study has two main strengths. The inclusion of two countries, Saudi Arabia and Egypt, which enhances the study’s external validity and provides a more comprehensive understanding of the phenomenon across different cultural contexts. Moreover, the study employed a validated questionnaire, ensuring the trustworthiness of the results. However, certain limitations should be considered. First, the use of convenience sampling via online platforms may introduce bias, as those who voluntarily participate might not accurately represent the broader population. Self-selection and the absence of randomization can introduce bias into online surveys via convenience sampling, thereby reducing generalizability. Researchers guaranteed survey transparency and targeted diverse populations in an effort to reduce bias. Furthermore, validated instruments were employed to enhance the dependability and accuracy of the outcomes derived from the online survey. Moreover, the use of an online survey may introduce selection bias, as it requires computer literacy. This could justify the low number of elderly participants in this study [17]. Second, ethical considerations and patient autonomy were not studied in this study. Addressing these ethical principles is essential when implementing strategies to address patients’ fears and concerns.

## Conclusions

Our study highlighted the high prevalence of needle fear within an adult population across large countries in the Middle East (Egypt and Saudi Arabia). Females, widowed, those with higher education degrees, unemployed, working in the health sector, and people with low income were more likely to have needle phobia. Medical practitioners should clearly and patiently explain procedures and implement several strategies that may help to have a remarkable effect on the patient’s fear. Our study results may also lay the groundwork for future research aimed at assessing and evaluating different strategies that overcome needle phobia, with the goal of improving patient outcomes.

## Data Availability

The datasets used and/or analyzed during the current study available from the corresponding author on reasonable request.
